# Evidence for the mechanosensor function of filamin in tissue development

**DOI:** 10.1038/srep32798

**Published:** 2016-09-06

**Authors:** Sven Huelsmann, Nina Rintanen, Ritika Sethi, Nicholas H. Brown, Jari Ylänne

**Affiliations:** 1 Department of Environmental and Biological Science, and Nanoscience Center, University of Jyväskylä, Survontie 9C, FI40014 Jyväskylä, Finland; 2 Department of Physiology, Development and Neuroscience, and Wellcome Trust/Cancer Research UK Gurdon Institute, University of Cambridge, Downing Street, Cambridge CB2 3DY, UK

## Abstract

Cells integrate mechanical properties of their surroundings to form multicellular, three-dimensional tissues of appropriate size and spatial organisation. Actin cytoskeleton-linked proteins such as talin, vinculin and filamin function as mechanosensors in cells, but it has yet to be tested whether the mechanosensitivity is important for their function in intact tissues. Here we tested, how filamin mechanosensing contributes to oogenesis in *Drosophila*. Mutations that require more or less force to open the mechanosensor region demonstrate that filamin mechanosensitivity is important for the maturation of actin-rich ring canals that are essential for *Drosophila* egg development. The open mutant was more tightly bound to the ring canal structure while the closed mutant dissociated more frequently. Thus, our results show that an appropriate level of mechanical sensitivity is required for filamins’ function and dynamics during *Drosophila* egg growth and support the structure-based model in which the opening and closing of the mechanosensor region regulates filamin binding to cellular components.

The concept of mechanical regulation of tissue development, mechanotransduction^1^, has been proven in several experimental settings. In adult mesenchymal stem cells, the elastic modulus of the culture substrate regulates cell differentiation along different lineages^2^. In muscles, mechanical stretching generates various responses that affect cell proliferation and differentiation^3^. Different sensor molecules, including many cytoskeleton-linked proteins, elicit these cellular responses according to the mechanical cues they perceive. For instance, the actin filament - plasma membrane linkers talin and vinculin regulate the mechanical tuning of cell adhesion strength by sensing the ECM rigidity^4^,^5^. Various Z-line proteins^6^,^7^ and the sarcomeric ruler titin^8^ modulate the mechanical signaling in muscles. Filamins cross-link actin filaments and anchor them to membranes^9^, and pulling forces regulate protein-interaction sites within their C-terminal immunoglobulin-like domains^10^,^11^,^12^,^13^ (Fig. 1a). The C-terminal mechanosensor region (MSR) of filamin has two protein interaction sites that are masked by neighbouring sequences (closed conformation, Fig. 1b) and masking is released by small forces of 2–5 pN (open conformation Fig. 1b)^13^. In cell culture models, the cytoplasmic tails of integrin adhesion receptors preferentially bind to open filamins as indicated by reduction of the interaction decay time when myosin is active^14^. Rare mutations in humans and animal models demonstrate that filamins are involved in three-dimensional tissue morphogenesis and the maintenance of muscle integrity^15^,^16^,^17^,^18^,^19^,^20^. Here we test whether the mechanosensor function of filamins has a role within tissues.

## Results

**Figure 1:**
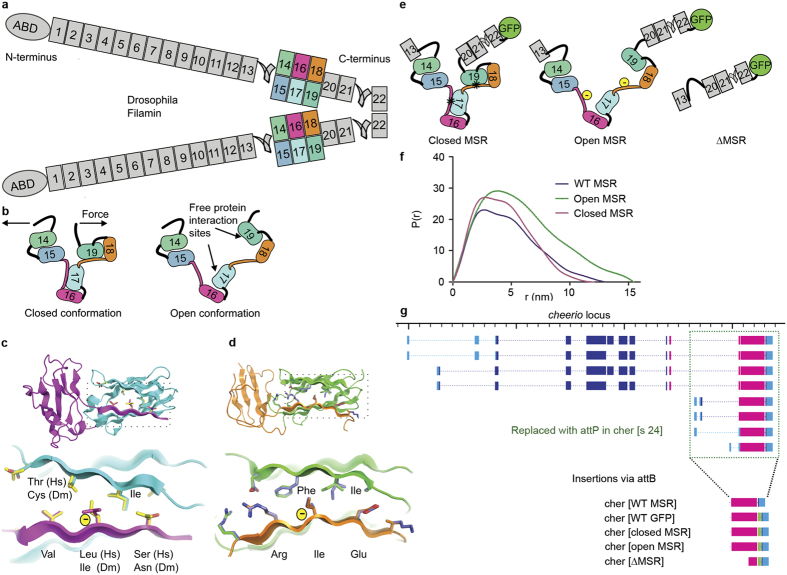
Cheerio, the conserved filamin of *Drosophila*. (**a**) A schematic of a Cheerio dimer. Numbered rectangles depict filamin immunoglobulin-like domains and coloured domains 14–19 comprise the mechanosensor region (MSR). Color code is maintained through the figure. Domain 22 is the dimerization site, ABD is the actin-binding domain. (**b**) Sketches of of the closed and the open conformation of the MSR. (**c**–**d)** Structures of the parts of human Filamin A corresponding to Cheerio domains 16–17 (**c**) and 18–19 (**d**) with the residues important for the masking interaction highlighted as sticks. The boxed region is shown enlarged in the lower images. Human amino acid residues are shown as yellow (**c**) or slate blue (**d**) sticks. Comparison of human (Hs) and Drosophila (Dm) sequences at these sites indicates that the main features of the masking interaction are well conserved. The yellow - symbols show the Ile and Leu residues substituted with Glu in the open MSR mutant. **(e)** Sketches of the mutants used in this study: the closed MSR mutant contains substitutions strengthening the masking interaction (marked with *), the open MSR mutant contains an added charge, the ΔMSR mutant lacks the MSR, but retains the dimerization site. (**f**) Particle distance distribution function P(r) of the purified five domain MSR fragments determined with small angle X-ray scattering. **(g)**The gene locus of *cheerio* indicating the deleted region (green dotted box) and newly integrated modified C-termini. Light blue boxes depict untranslated exons, dark blue exons encoding N-terminal (ABD to immunoglobulin-like domain 13) and magenta C-terminal parts of Cheerio (domains 14 to 22); green boxes indicate GFP(green fluorescent protein). (WT = wild type).

To test the role of filamin’s mechanosensitivity within intact developing tissues, we made new mutations in the gene encoding the the Drosophila filamin Cheerio. Sequence alignment with the corresponding parts of human Filamin A^10^ and structure-based homology modelling (Fig. 1c,d) suggest that the masking interactions of the C-terminal MSR are well conserved between human Filamin A and Cheerio. We used the structural information of the MSR from human Filamin A to generate Cheerio mutant proteins whose conformation would be shifted either to the open or the closed state. The open MSR mutant consists of Ile > Glu or Leu > Glu substitutions in each of the β strands that normally mask the two interaction sites (Fig. 1c–e). This mutation has been demonstrated to enhance integrin binding to human Filamin A^11^. The closed MSR mutation was generated by replacing the masking β strand in each site with a high affinity sequence from human platelet glycoprotein Ib^21^. This sequence has been shown to require higher forces, 6–12 pN, for the unmasking of the interaction site than the native sequence^13^. As consequence of these mutations, interactions with the MSR should require either greatly reduced mechanical pulling force (open mutation) or forces that are higher than normal (closed mutation). To see whether the mutants change the protein characteristics as expected, we measured the hydrodynamic behaviour of Cheerio MSR fragments (Ig-like domains 15–19) with or without the mutations. Small angle X-ray scattering analysis showed that the protein fragments behaved as single size particles in solution, and did not aggregate (Supplementary Fig. S1). The closed mutant had overall shape parameters similar to the wild type (WT) fragment (Fig. 1f and Supplementary Fig. S1), while the open mutant was more elongated, consistent with at least partial opening of the MSR even without external force.

**Figure 2:**
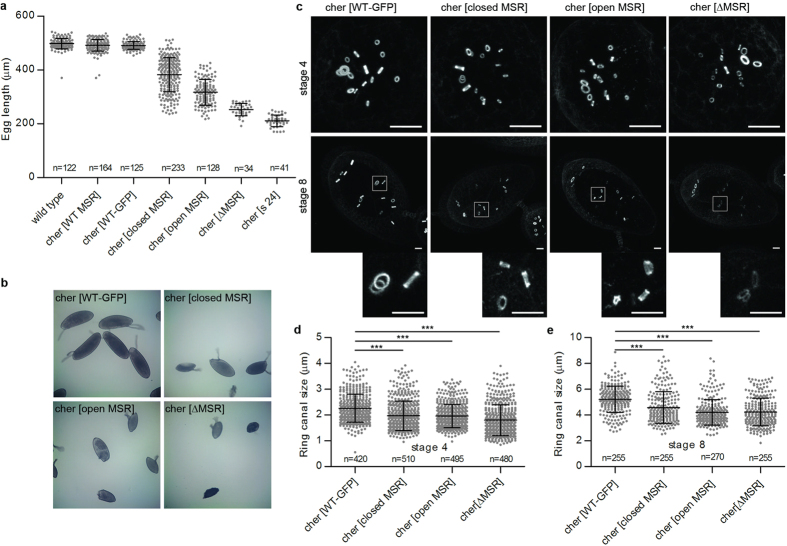
Mutating the mechanosensitivity of Cheerio disrupts egg development. (**a**) Length of eggs produced by hemizygous females. The C-terminal deletion (s24) and MSR deletion (ΔMSR) led to short eggs. The integration of the wild type construct with or without GFP (WT GFP and WT MSR, respectively) rescued completely the phenotype. Both mutations affecting the MSR sensitivity gave intermediate phenotypes (closed MSR and open MSR). Horizontal lines indicate the mean values and bars standard deviations The number of the eggs measured (n) is given. (**b**) Illustrative brightfield images of measured eggs. (**c**) All forms of Cheerio localised normally to ring canals, actin-rich rings, during early and mid oogenesis (homozygous stage 4 egg chambers, upper panels; stage 8 egg chambers, lower panels). Maximum intensity projections are shown. The boxed areas are shown below as enlarged images generated with the bicubic smoother option in Photoshop. Scale bars 10 μm. (**d,e)** Ring canal outer diameters of MSR mutants decreased in both stage 4 and 8 when compared to WT-GFP. Mean + SD, ***P < 0.0001, unpaired t-test.

To generate similar mutations within the endogenous *cheerio* locus of Drosophila, we next replaced the 3’ end of the *cheerio* gene with an attP recombination site by homologous recombination, and then reintroduced this gene segment in WT form, with or without a green fluorescent protein (GFP) tag, or with GFP-tagged mutant sequences: open, closed or ΔMSR (Fig. 1g). To restrict our analysis to the function of the long isoforms of filamin (Cher240) the promoter for the short Cher90 transcript was deleted in all constructs (which did not affect the processes examined here). Immunoblotting experiments showed that all alleles expressed comparable amounts of protein in flies and no major degradation products were observed (Supplementary Fig. S2). Consistent with the previous description of *cheerio* mutant phenotypes^22^,^23^,^24^, the C-terminal deletion (*cher*[s24]) caused sterile, small eggs (Fig. 2a). Restoring a single copy of the WT C-terminal coding sequence, with or without the GFP tag, fully rescued this phenotype (Fig. 2a,b). The ΔMSR allele led to almost as severe egg length phenotype as the C-terminal deletion. This demonstrates that the MSR is important for filamin function. The open and closed MSR mutations led to intermediate phenotypes (Fig. 2a,b), suggesting that correct mechanosensitivity of Cheerio has a functional role during oogenesis.

**Figure 3:**
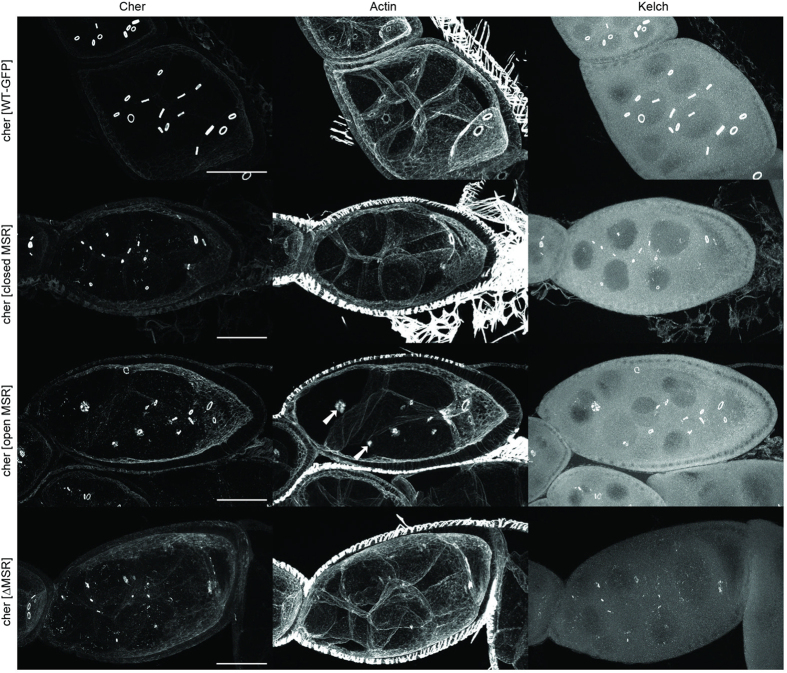
Perturbation of filamin’s mechanosensitivity alters ring canal actin but not localization of Kelch. Representative images of Cher-GFP, Rhodamine-Phalloidin (actin) and Kelch labelling of stage 8 chambers are shown. Images were taken from flies with hemizygous WT-GFT or MSR mutants. Note that when compared to the WT, at this stage many ring canals have disappeared in the ΔMSR mutant and some ring canals are smaller and fragmented in the open and closed MSR mutant. The arrows show ring canals that do not appear to be associated with membranes in the open MSR mutant. Scale bars 50 μm.

The small egg phenotype of *cheerio* mutants is caused by defects in the ring canal development blocking the transport of cytoplasm from nurse cells into the oocyte, the future egg^23^. A rapid growth of ring canals occurs between stages 5 to 10 of oogenesis^25^. This partially coincides (stages 8 to 10a) with rapid growth of the egg chamber during which the plasma membrane of nurse cells is under increased tension^26^. The deletion of Cheerio’s C-terminus caused smaller ring canals during early stages of oogenesis, followed by disappearance of ring canals from the stage 4 onwards, as observed for similar mutant alleles^22^,^23^,^24^. In contrast, in all our MSR mutants the ring canals only showed slightly reduced size at stage 4 (Fig. 2d), demonstrating that the mutant proteins retained substantial function. All components of ring canals that we tested (actin, HtsRC, Kelch, phospho-tyrosine) localised to the mutant ring canals at early stage (Fig. 3 and Supplementary Figs S3 and S4). However, defects in the function of the mutant filamins became apparent as development progressed. During stages 8 to 10a ring canals in the ΔMSR mutant remained small and often disappeared (Fig. 3, bottom row), while those in the open and closed MSR mutant remained small and thin, and sometimes fragmented at the same stages (Fig. 3, middle rows). In the open MSR mutant we often noticed membranes missing between nurse cells (14 out of 29 egg chambers scored, arrows in Fig. 3), whereas in the closed MSR mutant this was rare (2 out of 42 egg chambers). These observations show that the MSR mutants cause failure of ring canals during the time of increased tension of plasma membrane and occasionally cause disruptions of nurse cell plasma membranes. This suggests that Cheerio may have a role in sensing plasma membrane tension.

**Figure 4:**
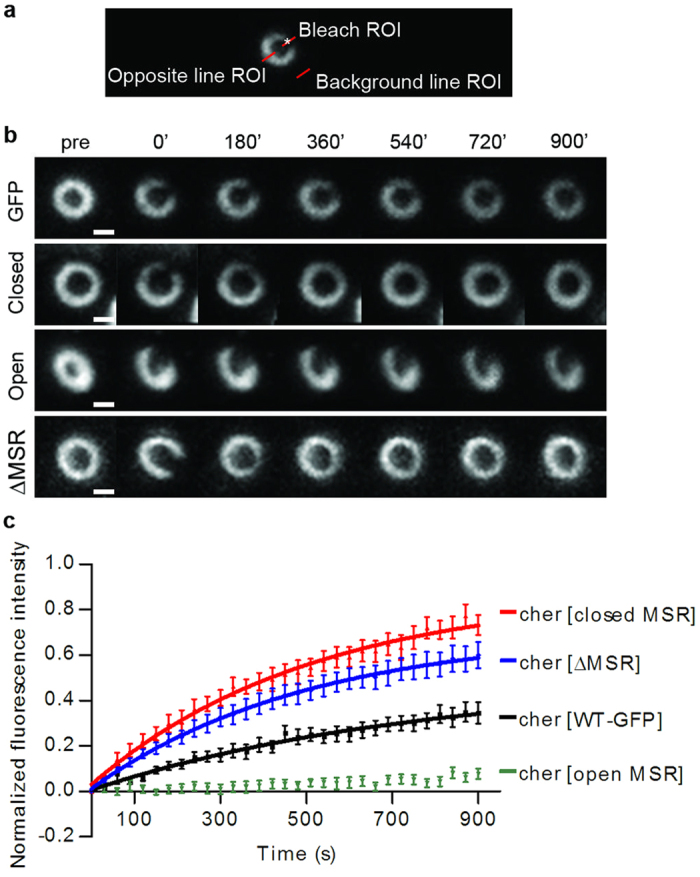
Cheerio mechanosensitivity affects protein dynamics *in vivo* (**a**) Setup of the fluorescent recovery after photobleaching (FRAP) experiments showing a single ring canal where the photobleached area is marked with a white asterisk and the three areas where the fluorescence signal was followed are indicated with red lines. (**b**) Representative maximum intensity projections of fluorescence recovery after photobleaching of Cheerio MSR mutated flies and WT. Scale bars 1 μm. (**c**) FRAP recovery curves of Cheerio WT and MSR mutants. The ΔMSR and closed MSR mutants are more mobile than the WT Cheerio. On the other hand, the open MSR mutant is less dynamic than WT.

The finding that Cheerio proteins with mutated or deleted MSR initially localised normally to ring canals, suggested that the MSR might regulate the dynamics of Cheerio, rather than its localisation. To test this with fluorescent recovery after photobleaching (FRAP), we bleached a small sector of a ring canal and followed the fluorescence recovery for 15 min (Fig. 4a,b). We found that Cheerio with the open MSR was less dynamic than the wild type control (Fig. 4b,c and Supplementary Movies S1, S2), whereas Cheerio with the closed MSR and ∆MSR showed increased recovery at ring canals compared to the wild type controls (Fig. 4b,c and Supplementary Movies S3, S4). Distinct exponential curves with similar recovery rates (Supplementary Table SI) could be fitted for WT, closed and ∆MSR mutant data points. In the case of the open MSR mutant, no exponential curve could be fitted due to poor fluorescence recovery. Thus, our results support the model in which myosin-mediated contractions, or membrane tension stretch actin-bound filamin, and result in the opening of interaction sites in the MSR^12^,^13^,^14^. In accordance with this, opening of the filamin MSR via mutations retained it more tightly at the ring canal and closing enhances its dynamics.

## Discussion

In this paper we show that filamin mechanosensing-altering mutations disrupt ring canal development in the Drosophila ovary and lead to small, partially sterile eggs. Of note, changing the spring properties of the mechanosensor site in either direction: releasing (open MSR mutation) or tightening (closed MSR), caused similar ring canal phenotypes, yet distinct dynamics of filamin in the ring canal. These data fit with a model in which filamins have a mechanosensory function during animal tissue development.

Definite mutations that alter spring properties of a cytoskeletal proteins have not been tested rigorously in animal models. For example, deletions in titin M-band region cause muscle development and regeneration defects in mouse^27^, but it is unclear to what extent altered mechanosensitivity contributes to these phenotypes. The unique structure of filamin MSR allowed us to make mutations that do not change the interaction sites themselves, but change how they are exposed. Previous structural and functional information allowed us to engineer mutations that either require less or more force for opening than the normal structure. The same mutations that we made here in the Drosophila filamin Cheerio have been earlier used in human Filamin A for biochemical studies^28^ and single molecule studies^13^. Our SAXS analysis suggested that the Cheerio MSR has similar shape parameters as the human Filamin A and that the open MSR mutation had changed the structure as expected. In the absence of external force the overall structure of the closed MSR mutant fragment was similar as WT.

For the *in vivo* experiments, we made the open and closed MSR substitution mutations in the genomic locus of *cheerio.* This allowed us to study the effects of mutations at normal gene copy number without interference from the WT gene. Although it has been previously shown that some mutations disrupting the structure filamins cause aggregation of the mutant protein in skeletal or cardiac muscle^20^,^29^ and that filamin unfolding may trigger chaperone-assisted autophagocytosis in muscle^30^, the MSR mutations used here did not cause filamin aggregates or decreased the protein amount. In contrast, immunoblotting experiments showed that all mutants were expressed at similar protein levels as the WT.

In addition to the finding that mechanosensing modifying filamin mutants disrupted the maturation of ring canals, the second main finding in the current study was that the mutants showed altered dynamics in the ring canal: open MSR mutant had markedly reduced recovery rate, whereas the closed MSR or ΔMSR mutant recovered faster than the WT filamin. This is consistent with the hypothesis that the open MSR interacts more strongly with ring canal components, whereas the interaction of closed MSR and ΔMSR mutants is mainly mediated by other regions of filamins, presumably the actin binding domain. Unfortunately, it was not possible to analyse the dynamics of the MSR mutants in the ring canals at late developmental stages, as the ring canals disintegrate in the MSR mutants.

How can we explain the apparently conflicting results that the open and closed MSR mutants cause similar ring canal phenotypes, but yet have dramatically different levels of exchange at their site of function? These observations do not fit with a strictly structural role of the filamin MSR in the ring canal. If that was the case, we would expect that the MSR interaction- stabilizing mutant would also abnormally stabilize the ring canal structure. We did not observe this. Instead, we favour a model in which different domains of filamin coordinate structural and mechanosensory roles during oogenesis. The C-terminal MSR region regulates the dynamics of filamins and is essential for maintaining the protein at ring canals. In contrast, the N-terminal actin-binding domain is not essential for localisation, but for ring canals growth: filamins with a non-functional actin binding domain localise to ring canals, but ring canals stay small^31^. Furthermore, our results suggest that to be fully functional the MSR must oscillate between open and closed conformations during ring canal maturation and growth: both, open and closed MSR mutants, destabilised the structure at the stage when it was supposed to grow. Thus, the data fit with a regulatory, mechanosensory function of filamin in ring canals. We would predict that growth of the ring canal would therefore involve membrane tension driven oscillations in the amount force within the structure. When under high tension filamin would bind tightly and perhaps also recruit new components to expand the structure. As the ring canal enlarges the tension would reduce, allowing filamin to redistribute within the expanded structure.

In mammalian model systems and in human patients, mutations in FLNA, FLNB and FLNC genes have been associated with developmental or regeneration abnormalities in vasculature, cartilage, bone and muscle^16^,^17^,^32^,^33^,^34^. Our current study suggests that some of these filaminopathies with either truncations of the C-terminal parts of filamins or point mutations at or near the MSR, may be caused by defects in mechanosensor function. For instance, missense mutations in the MSR of FLNC have been recently linked hypertrophic cardiomyopathy^19^.

In conclusion, our current results suggest that normal filamin function requires mechanically regulated conformational changes within developing intact tissues. This fits with the model that filamin has a mechanosensing function during tissue growth that is be conserved from flies to mammals.

## Materials and Methods

### Genomic engineering of Cheerio

The Cher C-terminal deletion and the knock-ins were generated according to Huang *et al*.^34^,^35^. The deletion removes the entire C-terminus including the promoter region of the short isoforms of Cher (between bp 17099072 and bp 17091407, chromosome 3R; FlyBase release r6.09) and inserts an attP site (Fig. 1d). For ends-out targeting we PCR amplified a 5 kb 5′homology arm and a 3.5 kb 3′homology arm using the genomic DNA (BAC RP98-2L16, BACPAC Resource center, Children’s Hospital Oakland Research Institute, Oakland, CA) as template, cloned them into pGX-attP^35^ via EcoR1 & Kpn1 and Spe1 & Xho1, respectively, and verified the amplified regions by sequencing. Two transgenic fly lines with pGX-attP insertions on the 2^nd^ chromosome were used to induce homologous recombination. From the potential *cher* knock-out line *cher*^s24w+^, which did not complement *cher* alleles *Df*(*3R*)*Ex6176* (Bloomington Drosophila Stock Center, Indiana University; Bloomington, IN) and *cher*^*EPSΔ5*^ 22,we removed the white marker using Cre^35^ and confirmed the engineered region by PCR. This line (*cher*^s24^) was then used as the founder line for all knock-ins. These knock-ins (*cher*^WT MSR^, *cher*^WT GFP^, *cher*^open MSR^, *cher*^closed MSR^, and *cher*^ΔMSR^) were generated by site-directed mutagenesis of the *cher* 3′ end (last five exons) from BAC RP98-2L16. The fragments were tagged with mGFP6^36^ and inserted into pGE-attB^GMR^^35^ via EcoRI and KpnI sites. All constructs were verified by sequencing, and integrated into the attP site of the founder line. Further details of cloning and genetics are available on request.

### Measurements of egg length

24–48 hrs old hemizygous *cher*^WT MSR^ or mutant females fed on fresh yeast were allowed to lay eggs for 16 hrs on apple juice agar plates. Eggs were collected, rinsed with water, mounted in 3S Voltalef oil, and imaged with 10 x objective on a Leica DMR microscope with a MarcoFire camera (Optronix). The egg length was measured from the images with Image J line tool.

### Immunofluorescence and Immunoblotting of egg chambers

Young females (up to 24 hrs old) were put on fresh yeast with males for 36 to 48 hrs. Ovaries were dissected and stained as described in^31^. We used the following primary antibodies: mouse anti-phospho-tyrosine (1:200, clone 4G10, Millipore); mouse anti-kelch kel-1B^37^ (1:1, Developmental Studies Hybridoma bank, DSHB, University of Iowa, Iowa, US) and mouse anti-HtsRC (1:10, DSHB). Used secondary antibodies and phalloidin are specified in^31^. After washes, ovarioles were mounted in Vectashield (Vector Laboratories) or Mowiol-Dabco (Sigma). Samples were imaged with a 60x oil objective on a FV-1000 (Olympus) or A1R (Nikon) confocal microscope by using sequential imaging. For immunoblotting we used denatured protein extract from five sonicated ovaries for each genotype. Rabbit anti-GFP (1:2000, Abcam, UK) and rat anti-Ct-FLN (1:4000, a gift from L. Cooley) were primary antibodies, Goat Anti IgG (H&L) Rabbit DyLight 800 (1:8000, tebu-bio, UK) and Goat anti IgG (H&L) Rat DyLight 680 (1:8000, tebu-bio, UK) were secondary antibodies. Images were taken on a Odyssey system from Licor Biosciences (Cambridge, UK).

### Small angle X-ray scattering

The region of domains 15–19 of Cheerio was PCR amplified from the corresponding pGE-attB-Cher plasmid as templates and cloned into the GST-fusion protein vector pGTVL1 (Structural Genomics Consortium, University of Oxford) using the ligation independent cloning method. The short intron sequence within the fragment was removed using QuikChange site-directed mutagenesis kit (Agilent Technologies). All PCR products were confirmed by sequencing. The three constructs pGTVL1-Cher15-19wt, pGTVL1-Cher15-19closed, and pGTVL1-Cher15–19open were then transformed into the *Escherichia coli* BL21 Gold strain (Agilent Technologies) and the recombinant proteins were expressed at 37 °C for 4 hrs in the presence of 0.4 mM Isopropyl β-D-1-thiogalactopyranoside (IPTG).The proteins were first purified with Glutathione Agarose 4B affinity column (Protino, Machery Nagel) and the GST fusion partner was then cleaved using Tobacco Etch virus protease (Invitrogen, Life technologies). Final purification step was done with gel filtration using HiLoad 26/60 Superdex 75 column (GE Healthcare) in buffer containing 100 mM NaCl, 1 mM dithiothreitol (DTT) and 20 mM Tris pH 7.5. Finally, proteins were concentrated using Centriprep YM-10000 centrifugal concentration devices (Millipore).

Small angle x-ray Scattering (SAXS) experiments were performed at 10 °C at the P12 beamline at the European Molecular Biology Laboratory (EMBL), Hamburg (Pilat us 2M detector, sample-detector distance of 3.1 m, and wavelength λ = 0.124 nm). Four protein dilutions were used in the range of 1–10 mg/ml with 10 mM DTT added to the purification buffer, to check for concentration dependent scattering. Buffer scattering was subtracted from the sample scattering. The analysis of the scattering data and model building was done as described in^38^ using programs of the ATSAS package^39^.

### Egg chamber culture and live fluorescence microscopy

Egg chambers were dissected in Schneider’s Drosophila medium (Gibco) supplemented with 5% Fetal Calf Serum (Gibco) and 5 μg/ml insulin (Sigma). For imaging, media was further supplemented with 2 mg/ml trehalose (Sigma), 50 ng/ml adenosine deaminase (Roche), 5 μM methoprene (Sigma) and 1 μg/ml ecdysone (Sigma). Intactness of membranes and health of egg chambers were controlled by adding 8.3 μM FM6-46 (Invitrogen) to imaging solution. Dissected egg chambers were imaged in Poly-D-Lysine coated (1 mg/ml, Sigma) LabTek chambered goverglasses (Nunc) at RT. GFP-tagged Cheerio was imaged with Nikon A1R confocal microscope and 60x/1.2 WI objective. 488 nm Argon laser excitation and 515/30 nm emission filter were used for the GFP.

For ring canal size measurements, living, stage 4 or stage 8 egg chambers were imaged with 1 μM interval, pinhole size 33.2 μm, Nikon A1R confocal microscope. Egg chambers in diameter of 40–55 μm were classified as stage 4 egg chambers and stage 8 chambers had visible yolk, uniform follicle cell layer and oocyte size less than 1/3 of the egg chamber volume^40^. Outer diameter of ring canals was measured from maximum intensity projections of Cheerio-GFP signal by using ImageJ line tool and measure function. 28 to 35 egg chambers were imaged in each case. Statistical analysis was done with unpaired, two-tailed Student’s t test with Prism 5 software (GraphPad Software Inc).

For FRAP Z-stacks with 8 μM range at 0.23 μm interval were imaged. After one pre-bleach image stack, point bleaching was done with 405 nm 100 mW laser with 100% power for 5 ms at the middle section and 31 after bleach z-stacks were taken with 30 s interval. Individual bleached ring canals were cropped with ImageJ and registered in x-y direction with PoorMan3DReg plugin (written by Michael Liebling and relying on Philippe Thévenaz’s TurboReg and Charlie Holly’s Grouped ZProjector plugins). From registered ring canals the intensity of bleached, unbleached and background areas were measured by using line tool and Multi Measure tool of ROI manager of ImageJ. To normalize FRAP data, equation (1) was used,


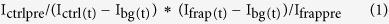


where *I*_*ctrl pre*_ is the fluorescence intensity of control line in prebleach image, *I*_*ctrl* (*t*)_ is intensity of ctrl line at time point t and *I*_*bg* (*t*)_ is the intensity of background at timepoint t. Similarly, *I*_*frap* pre_ or I_frap (*t*)_ are the intensity of point bleached area in prebleached image and at time point t, respectively. Shortly, background was subtracted from raw data and fluorescence loss due to imaging was taken into account by normalization with unbleached area of the same ring canal. Finally, to compare results of different mutants the data were re-normalized by setting the after bleach intensity to zero. 15 ring canals of stage 4 egg chambers were analysed from *cher*^WT GFP^, *cher*^*c*losed MSR^ *cher*^open MSR^ and *cher*^*Δ*MSR^ homozygous flies with Prism 5 software (GraphPad Software Inc). Each data point in graph represents the mean of the fluorescence intensity at the time point. Single exponential curve, equation (2), was fitted and data was subjected to non-linear regression analysis.


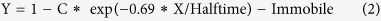


## Additional Information

**How to cite this article**: Huelsmann, S. *et al*. Evidence for the mechanosensor function of filamin in tissue development. *Sci. Rep.* **6**, 32798; doi: 10.1038/srep32798 (2016).

## Supplementary Material

Supplementary Information

Supplementary Video S1

Supplementary Video S2

Supplementary Video S3

Supplementary Video S4
