# Supplementary material for: Evidence for the mechanosensor function of filamin in tissue development
Source: Sci Rep. 2016 Sep 6;6:32798. doi: 10.1038/srep32798 (PMC5011733; doi:10.1038/srep32798)
Supplement: Supplementary Information [file srep32798-s1.pdf]

Evidence for the mechanosensor function of filamin in tissue development

Sven Huelsmann, Nina Rintanen, Ritika Sethi, Nicholas H. Brown, and Jari Ylänne

Supplementary Materials:

Figures S1-S4

Table S1

Captions to Movies S1-S4

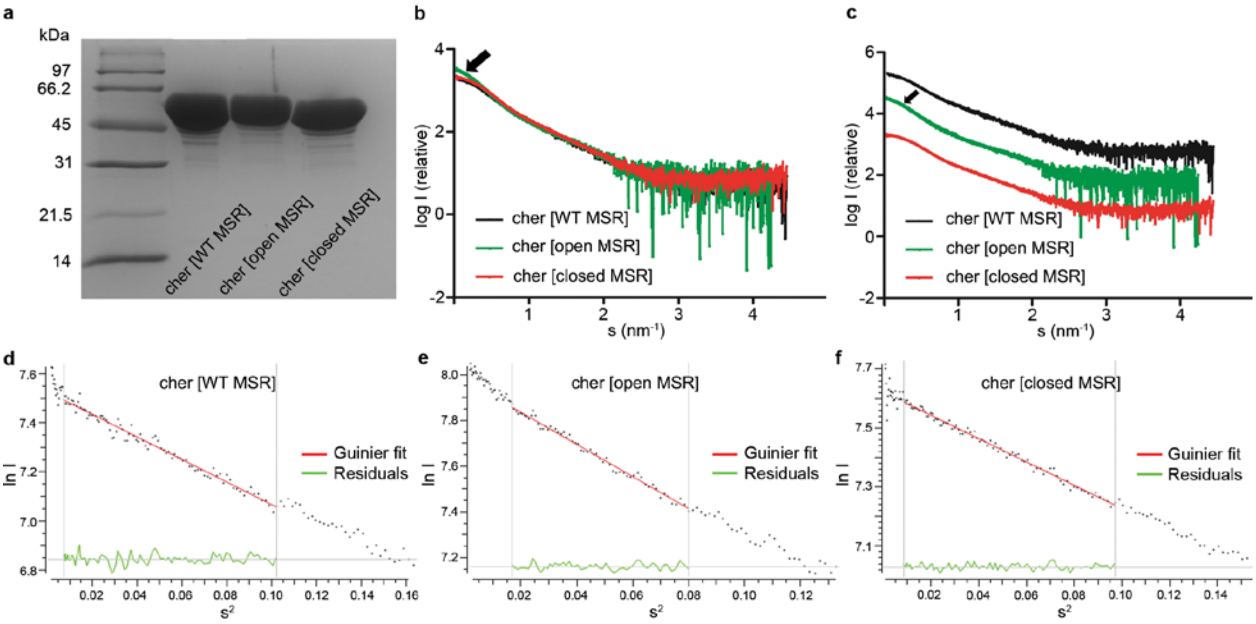

Figure S1. Biochemical and biophysical characterization of the five domain Cheerio fragments. (***a***) SDS-PAGE analysis of the purified five domain fragments. (**b-c**) Small angle X-ray scattering (SAXS) intensity (I) measured from the purified five domain fragments given as function as momentum transfer s ($s=4\pi\sin\theta/\lambda$ where 2θ is the angle between the incident and scattered radiation). In (**c**) the scattering curves are nudged by 1 log unit to allow better comparison of the curve features. The arrow indicates the feature in the scattering profile that distinguishes Cher [open MSR] construct from the others. (**d-f**) Guinier analysis of the scattering data shown in b. The experimental scattering values for the low s region is shown with black dots. The Guinier fit is shown in red. The residual differences between the fit and the data points are shown in green on the below the fitted area. As the residuals show no trend below or above the fit, the analysis is consistent with the particles being monodisperse.

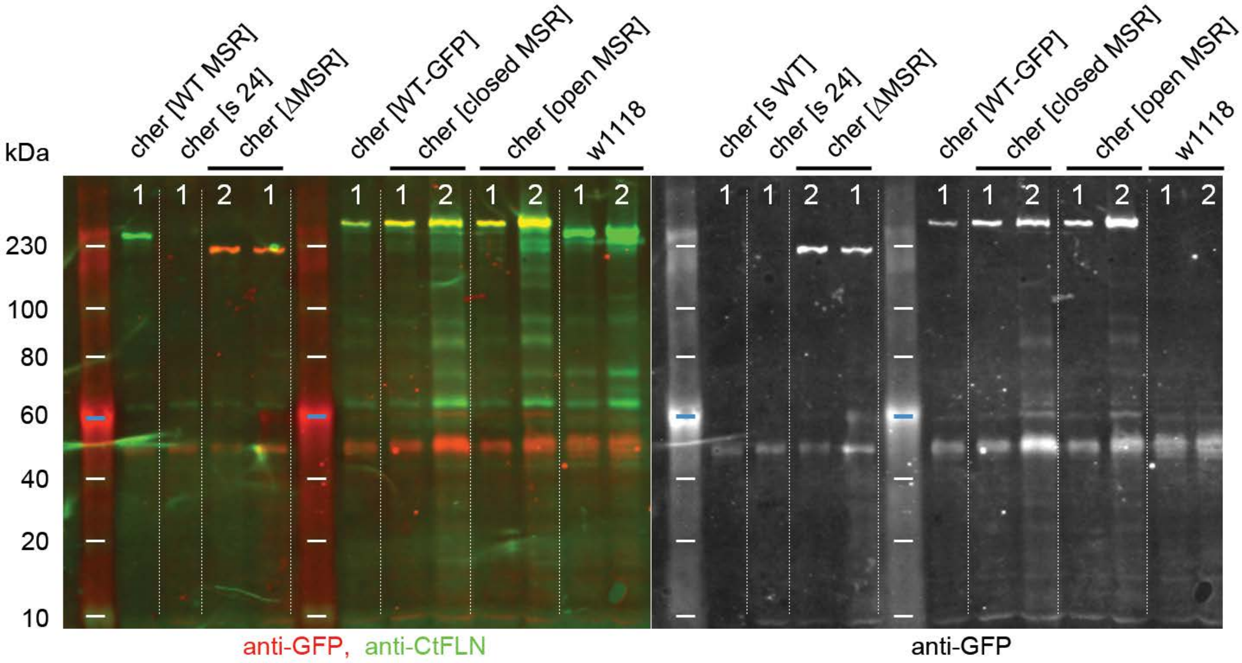

Figure S2. Western analysis of Cheerio protein levels in ovaries. Fixed amount of protein was run on SDS-PAGE from ovaries of females carrying either 1 copy (with deficiency *Df(3R)Exel6176*) or 2 copies of the *cher* alleles and probed with antibodies recognising the C-terminus of Cheerio (green), and GFP (red). The left panel shows the green and red channels overlaid and the right panel show the red channel alone in grayscale. This experiment confirms that neither Cheerio s24 protein nor the Cheerio ΔMSR protein is recognized by the CtFLN antibody. This analysis also shows that in each case two gene copies of the alleles produce more protein than a single copy and the expression levels of the mutants are comparable to the wild type. Comparison of the multiple bands observed with the CtFLN antibody shows no consistent differences with the WT or mutant Cheerio.

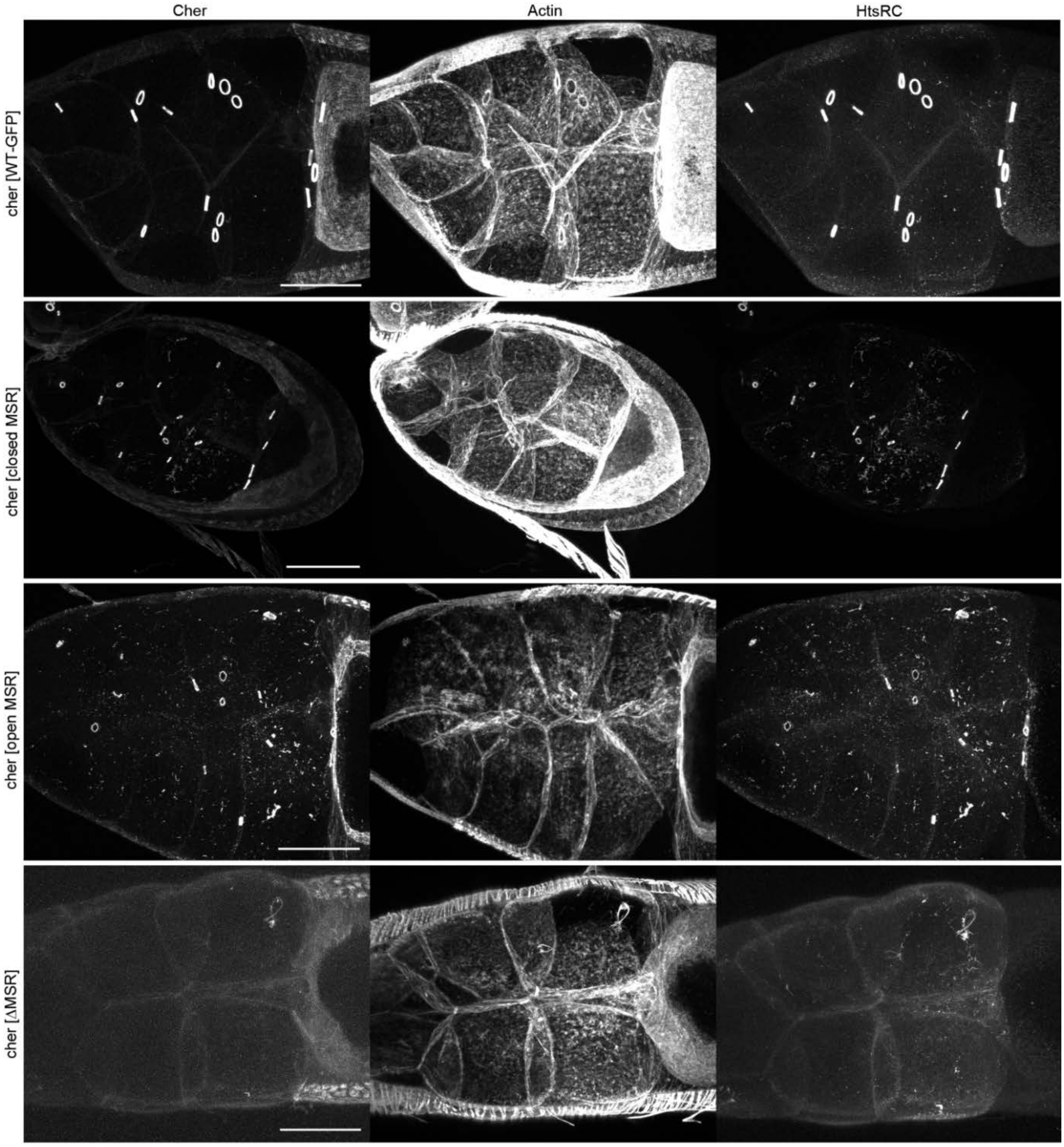

Figure S3. MSR region mutations affect the integrity of ring canals at late oogenesis. Representative images of Cher-GFP, Rhodamine-Phalloidin (actin) and HtsRC labeling of stage 9 or 10A egg chambers. Images taken from flies with WT or mutant Cher-GFP over deficiency *Df(3R)Ex6186*. Note the small, faint and fragmented ring canal structures particularly in the ΔMSR mutant. Scale bar 50 μm.

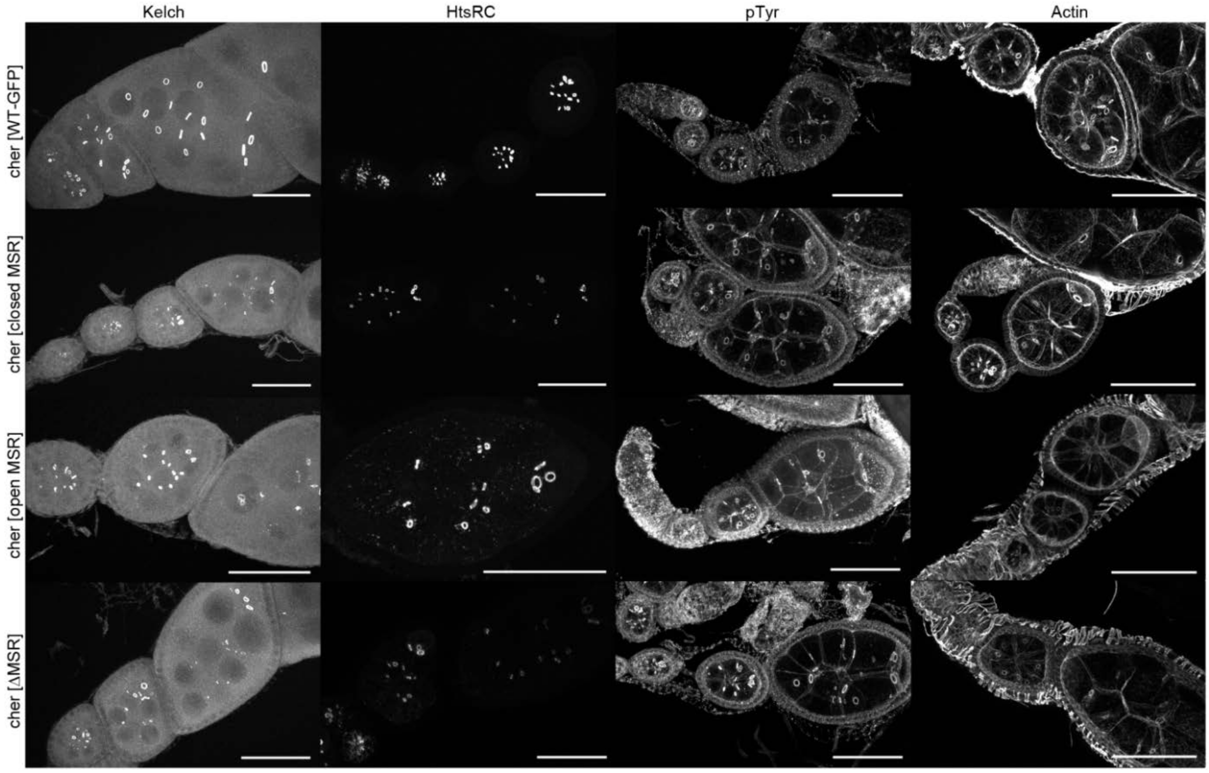

Figure S4. Ring canal proteins localize normally in Cheerio MSR mutant flies. Representative images of egg chambers are shown stained individually for the indicated markers. Kelch and HtsRC labeling of WT or mutant Cher-GFP over deficiency (*Df(3R)Exel6176*) flies and phospho-tyrosine and actin labelling of homozygous Cher-GFP flies are shown. These images show that during early oogenesis all the tested markers can be detected at the actin-rich ring canal structures in mutant ovaries. Scale bar 50 μm.

Table S1 **Halftimes and immobile fractions of WT and mutant Cheerio from FRAP analysis**

	Half-time ± s.e. (s)†	immobile fraction ± s.e.
Cher [WT GFP]	529.2 ± 188.0	0.507 ± 0.102
Cher [closed MSR]	355.7 ± 59.24	0.121 ± 0.066
Cher [open MSR]	not determined*	not determined
Cher [ΔMSR]	348.9 ± 70.25	0.295 ± 0.065

†Half-times and immobile fraction were determined from single exponential curve fits from 15 stage 4 egg chambers in each case. * In case of Cher [open MSR], exponential curve could not be fitted due to lack of recovery

Movie S1

Fluorescent recovery after photobleaching of Cheerio with WT MSR

This video complements Figure 4. Maximum intensity projection of photobleached ring canal from cher [WT-GFP] homozygous fly is shown. Photobleaching was done after the first frame and recovery of fluorescence is followed with 30 s interval for 15 min. 290x sped up.

Movie S2

Fluorescent recovery after photobleaching of Cheerio with open MSR

This video complements Figure 4. Maximum intensity projection of photobleached ring canal from cher [open MSR] homozygous fly is shown. Photobleaching was done after the first frame and recovery of fluorescence is followed with 30 s interval for 15 min. 290x sped up

Movie S3

Fluorescent recovery after photobleaching of Cheerio with closed MSR

This video complements Figure 4. Maximum intensity projection of photobleached ring canal from cher [closed MSR] homozygous fly is shown. Photobleaching was done after the first frame and recovery of fluorescence is followed with 30 s interval for 15 min. 290x sped up

Movie S4

Fluorescent recovery after photobleaching of Cheerio with deleted MSR

This video complements Figure 4. Maximum intensity projection of photobleached ring canal from cher [ΔMSR] homozygous fly is shown. Photobleaching was done after the first frame and recovery of fluorescence is followed with 30 s interval for 15 min. 290x sped up
